# Investor Behavior and Flow-through Capability in the US Stock Market

**DOI:** 10.3389/fpsyg.2016.00668

**Published:** 2016-05-09

**Authors:** Carlos Cano, Francisco Jareño, Marta Tolentino

**Affiliations:** ^1^Department of Economic Analysis and Finance, University of Castilla-La ManchaAlbacete, Spain; ^2^Department of Economic Analysis and Finance, University of Castilla-La ManchaCiudad Real, Spain

**Keywords:** flow-through capability, inflation rate, stock return, sectoral analysis, investor behavior

## Abstract

This paper analyzes investor behavior depending on the flow-through capability (FTC) in the US stock market, because investors seek protection from inflation rate changes, and the FTC (a firm's ability to transmit inflation shocks to the prices of its products and services) is a key factor in investment decisions. Our estimates of the FTC of firms listed on the US stock exchange at the sector level are significantly different among industries, and we demonstrate a direct relationship between changes in stock prices (at the sector level) and FTC. These results would be relevant because they have important implications on investor behavior.

## Introduction

Campbell (2006), Sekscinska (2015), and González et al. (2016), among others, point out that investor behavior is concerned about managing the economic and financial risk. To that end, a measure of the firm's ability to transmit inflation shocks to the prices of its products and services could be really relevant for investors and portfolio managers. Thus, the main objective of this research is to estimate the capability of American companies to transmit inflation shocks to the prices of the products that they sell and/or the services that they provide (Asikoglu and Ercan, 1992; Jareño and Navarro, 2010) and to identify any significant differences among sectors, because the investor behavior may be quite different depending on this flow-through capability.

Flow-through capability (FTC) is defined as a firm's ability to transmit inflation shocks to the prices of its products and services. The concept of “FTC” was introduced by Estep and Hanson (1980), but Asikoglu and Johson (1986, 1990) and Asikoglu and Ercan (1992) were the pioneers in analyzing the negative relationship between inflation and stock returns in the United States at the sector level. The authors conclude that the negative effect of an increase in the inflation rate on the stock price of a company is inversely related to the company's capability to transmit inflation shocks to its prices.

Empirical evidence (Jareño, 2005; Jareño and Navarro, 2010) suggests that FTC has an effect on stock prices and that there are significant differences in FTC at the sector level. Namely, industries in which the flow-through (FT) coefficients are greater exhibit stock prices that are less sensitive to inflation shocks. Therefore, increments in FT capability are linked to increments in stock prices.

Jareño and Navarro (2010), studying the Spanish stock market, obtain evidence of a strong negative relationship between the sensitivity of stock returns to changes in nominal interest rates and the ability to absorb inflation. Specifically, absorption ability can explain ~50% of the differences in stocks' sector durations in the face of changes to nominal interest rates.

Other studies, such as Ertek (2009), are based on the concept of FT capability and conclude that the higher the percentage of inflation shocks that translate into growth in a company's profit rate, the greater the company's stock price (and vice versa). In addition, Ertek (2009) incorporates FT capability into a quantitative model for stock selection to create quality portfolios with inflation hedge in the European sphere. These portfolios are built by selecting individual companies based on a series of characteristics believed to affect returns provided by different assets, considered to be linked to inflation hedge.

Given that related literature is scarce, we highlight this study's contributions. First, we use quarterly data, as opposed to the semiannual data used in previous studies. Second, we propose an alternative method of measuring the ability to absorb inflation, using a proxy variable that is different from the production level. Third, we confirm, for the sample analyzed, a positive relationship between changes in stock prices and companies' FT capability at the sector level, in agreement with Asikoglu and Ercan (1992). Therefore, we find that investor behavior may be quite different according to the FTC of the sector of activity that each company belongs to.

The remainder of the study is structured as follows. Section Estimation of the FTC describes not only the data but also the methodology that was proposed and employed. Section The Relationship between FT Coefficients and Stock Prices shows how we estimated companies' ability to absorb inflation, as analyzed by sector in the US stock market. The primary results are gathered and interpreted in Section Overall Results. Finally, Section Discussion highlights this study's primary conclusions.

## Materials and methods

This study assumes that the investor behavior may be different depending on the FTC, because investors want to protect from interest and inflation rate changes (González et al., 2016), so the FTC is a relevant factor in investment decisions. Moreover, according to Asikoglu and Ercan (1992) and Jareño (2005), among others, companies characterized by higher FT capability would show higher stock prices. As a result, in sectors with higher FT coefficients, stock prices would be less sensitive to inflation shocks.

Moreover, Kusev and van Schaik (2011), and Sekscinska (2015), among others, assume that financial and economic decisions, considered by previous investors as good or bad ones, may affect risk preferences, so prior experiences could determine their subsequent decisions. Blackburn et al. (2014) also find that investor decisions may depend on past returns. Therefore, the analysis of the FT capability would be really crucial for investors because it enables them to better manage interest and inflation risk.

Thus, this research is based on the methodology proposed by Jareño (2005) and Jareño and Navarro (2010) as applied to the United States, in order to estimate this relevant FT capability of each company depending on the sector belongs to. We used quarterly data, which is a marked improvement compared to previous studies that primarily used semiannual data. The study period is 2000 through 2009, and 40 observations were obtained.

In Jareño (2005), which the starting point of this research, the FT coefficients of the Spanish case are estimated, taking as a reference semiannual data from 150 companies traded in the stock market during the sample period, classified by activity sector (according to the classification established by the Madrid stock market).

This study begins by proposing the following theoretical model:

(1) $$\begin{array}{l} △V_{t}=p_{t+1}\cdot \;q_{t+1}-p_{t}\cdot q_{t} \end{array}$$

where *V*_*t*_ is company sales during period t, *p*_*t*+1_ is the mean price of products sold by the company during period t+1 and *q*_*t*+1_is the mean production (in physical units) during period t+1.

However, data related to goods sold and/or services provided are not available, which is why we used a *proxy* variable, namely, the number of employees. According to Jareño and Navarro (2010), this variable allows a good approximation if we assume constant productivity. Because the number of years in the sample is not very high, we assume that hypothesis. One important contribution of this research is to propose an alternate proxy variable from that used by Jareño (2005) and Jareño and Navarro (2010); with that alternative proxy variable, it is possible to make a second estimation of the FT capability of North American companies at the sector level.

Substituting production in Equation (1) with its equation according to productivity and the number of employees, we obtain the following formula:

(2) $$\begin{array}{l} △V_{t}={△p}_{t}\left(\omega_{t+1}{pdd}_{t+1}\right)+p_{t}{pdd}_{t}{△\omega }_{t}+p_{t}\omega_{t+1}q_{t}{△pdd}_{t} \end{array}$$

where ω_*t*+1_ is the mean number of employees in t+1 and *pdd*_*t*+1_ is the productivity for each employee. Assuming constant productivity, we would obtain the following equation:

(3) $$\frac{\Delta V_{t}}{V_{t}}=\frac{\Delta p_{t}}{p_{t}}\left\lfloor \frac{\omega_{t\text{+1}}-\omega_{t}}{\omega_{t}}\text{+1}\right\rfloor +\frac{\Delta \omega_{t}}{\omega_{t}}=\frac{\Delta p_{t}}{p_{t}}\frac{\Delta \omega_{t}}{\omega_{t}}+\frac{\Delta p_{t}}{p_{t}}\frac{\Delta \omega_{t}}{\omega_{t}}$$

The relative increase in company sales is equal to the sum of two terms and their cross product. If we assume that the latter is negligible, we obtain the following simplified equation:

(4) $$\begin{array}{l} \frac{△V_{t}}{V_{t}}\approx \frac{{△p}_{t}}{p_{t}}+\frac{△\omega_{t}}{\omega_{t}} \end{array}$$

The annual growth of the sales variable is calculated as the quarterly payment with respect to the same payment for the previous year, thus avoiding seasonal problems.

(5) $$\begin{array}{l} \frac{\Delta V_{t}^{t-4}}{V_{t-4}}=\frac{V_{t}-V_{t-4}}{V_{t-4}} \end{array}$$

where *V*_*t*_ is the business net turnover during the quarterly period “t”.

Conversely, there is no data available for variable*p*_*t*_, and thus we assume that the increase in prices for the company's goods and services sold/provided have a linear relation to the economy's previous inflation period:

(6) $$\begin{array}{l} {△p}_{t}=f\left(△{IPC}_{t},△{IPC}_{t-1},\ldots \right)=\alpha_{0}△{IPC}_{t}+\alpha_{1}△{IPC}_{t-1}+\ldots \end{array}$$

where *IPC*_*t*_ is the US consumer price index in period t, and α_0_, α_1_ are the FT coefficients (such as the Spanish coefficients estimated by Jareño, 2005).

Considering Equation (6) in (4), the general theoretical model proposed by Jareño (2005) and Jareño and Navarro (2010) is as follows:

(7) $$\begin{array}{l} \frac{\Delta V_{t}^{t-4}}{V_{t-4}}=\beta_{0}+\beta_{1}\frac{\Delta \omega_{t}^{t-4}}{\omega_{t-4}}+\beta_{2}\pi_{t}^{t-4}+\varepsilon_{t} \end{array}$$

where *V*_*t*_ is the “sales” variable for quarterly period t, ω_*t*_ refers to the mean number of employees in t, Δ*V*${}_{t}^{t-}$^4^/*V*_*t*−4_ is the growth rate of the “sales” variable, Δω${}_{t}^{t-}$^4^/ω_*t*−4_ represents the growth rate of the *proxy* variable of production, $\pi_{t}^{t-2}$ is the year-on-year inflation rate, β_0_, β_1_, and β_2_ show the parameters of the model and ε_*t*_ is the error term, which follows a normal distribution with zero mean.

According to Equation (7), to estimate FT capability, two exogenous variables are included, assuming variations in sales of the company can be caused by fluctuations in the inflation rate and the company's production level:

- Inflation rate is calculated using the quarterly average of the retail price index with respect to the same semester for the previous year, considering that *p*_*t*_ is the level of prices during month t. In our case, the frequency is quarterly instead of semiannually, which means greater precision in estimating the FT coefficients.
- Production level is obtained in the same manner as the sales variable:
  (8) $$\begin{array}{l} \frac{\Delta \omega_{t}^{t-4}}{\omega_{t-4}}=\frac{\omega_{t}-\omega_{t-4}}{\omega_{t-4}} \end{array}$$

where ω_*t*_ is the mean number of employees during quarterly period t. That notwithstanding, this paper presents a proposal to approximate the production level.

Conversely, Leibowitz et al. (1989) assume that the growth of a company's return rate can be approximately measured using the following equation, as assumed by Jareño and Navarro (2010):

(9) $$\begin{array}{l} g\approx g_{0}+\gamma r+\lambda \pi \end{array}$$

where *g* is the growth rate of profits—equivalent to *g* in the simplified equation of the dividend discount model by Gordon (1959) and Gordon and Shapiro (1956), *r* is the real interest rate, π represents the expected inflation rate, *g*_0_ is a constant that represents the long term growth rate, γ denotes the sensitivity of the growth rate of future profits to changes in the real interest rate and λ is the FT coefficient.

Following the logic of Jareño and Navarro (2010), the FT capability is related to a company's ability to translate inflation shocks into increased product/service prices, namely, Δ*p*_*t*_ [as shown in Equation (6)]. Specifically, we assume that:

(10) $$\begin{array}{l} \frac{△p_{t}}{p_{t}}=f\left(\pi_{t},\pi_{t-1},\ldots \right)=\alpha_{0}+\alpha_{1}\pi_{t}+\alpha_{2}\pi_{t-1}+\cdots +u_{t} \end{array}$$

where α_*i*_ measures the company's ability to transmit such shocks (previous ones and current ones) to the prices of its products, that is, it represents the essence of the FT coefficient concept, the estimation of which is one of this study's primary objectives.

This way, combining Equations (4) and (10) results in the following relationship, where*e*_*t*_ is the error term:

(11) $$\begin{array}{l} \frac{△V_{t}}{V_{t}}=\alpha_{0}+\alpha_{1}\pi_{t}+\alpha_{2}\pi_{t-1}+\cdots +\delta \frac{{△\omega }_{t}}{\omega_{t}}+e_{t} \end{array}$$

Thus, to relate Equation (11) with the equation provided by Leibowitz (9) and the FT coefficient, we assume that the growth of the return rate of company (*g*) depends on both the relative changes in its turnover and on other variables that we denote as $\bar{\theta }$ (omitted variables such as technological changes and other macroeconomic factors):

(12) $$\begin{array}{l} g=f\left(\frac{\Delta V_{t}}{V_{t}},\bar{\theta }\right)=f(\alpha_{0}+\alpha_{1}\pi_{t}+\alpha_{2}\pi_{t-\text{1}}+\ldots \\ \;+\;\delta \frac{\Delta \omega_{t}}{\omega_{t}}\;+\;e_{t},\bar{\theta }) \end{array}$$

In addition, if we assume that *f* is linear with respect to π, we can write:

(13) $$\begin{array}{r} \text{g}=m\left(\beta_{0}+\beta_{1}\frac{{△\omega }_{t}}{\omega_{t}},\bar{\theta }\right)+\emptyset_{1}\pi_{t}+\emptyset_{2}\pi_{t-1}+u_{t} \end{array}$$

Assuming that *E*_*t*_[*u*_*t*_] = 0 and taking into account Equations (13) and (9), we arrive at the following:

(14) $$\begin{array}{r} g_{0}+\gamma r\approx m\left(\beta_{0}+\beta_{1}\frac{{△\omega }_{t}}{\omega_{t}},\bar{\theta }\right) \end{array}$$

This way, the growth of a company's return rate over the long term and the real interest rate are related to its economic situation and indirectly to increases in the labor force and other residual variables, such as technological changes and other macroeconomic factors ($\bar{\theta }$).

The remaining terms in Equation (13), ϕ_1_π_*t*_ + ϕ_2_π_t−1_, are related to FT capability (πλ). Specifically, Jareño and Navarro (2010) assume that Φ is monotonically related to λ, thus demonstrating a negative relationship between FT capability and the duration of stocks equivalent to a negative relationship between parameters Φ_*i*_ and the sensitivity of stocks to changes in the nominal interest rate.

This relationship is really important, because this connection would point out the relevance of this study for investor behavior (Blackburn et al., 2014; González et al., 2016). Thus, if this research could find significant differences in the FTC of companies by sector, then investors would seek protection from interest and inflation rate changes by taking into account this FT capability as a key factor in their investment decisions.

## Estimation of the FTC

### Data and sector classification

To estimate the ability to absorb inflation (or FT ability) in the context of the United States, we use as a starting point Equations (7) and (11). Following this logic, we use individual data from 500 companies traded on the S&P 500. In addition, we classify the companies first by subsectors and then by activity sectors.

In addition, the study period is 2000–2009 (inclusive) and the frequency of data is quarterly, leading to 40 observations. The data were extracted from public reports of balance sheets and profit-and-loss statements, specifically, the quarterly sales of companies in the S&P 500, the number of employees in each quarter and the alternative proxy variable of production for each company. Some examples that support the selection of this proxy variable are Everaert and De Simone (2007), He (2010), Marple (1986), Rodrigues and Brady (1992), and Staunton (1986). Finally, we used data on the United States' inflation rate.

The United States inflation rate was obtained through data supplied by the Harmonized Consumer Price Index (for 2005), published by Eurostat. This way, we first calculated the inter-annual inflation rate with a monthly frequency to eliminate seasonal problems. Then, we converted the data to a quarterly frequency so that it coincided with the rest of the variables: i.e., turnover of the companies listed in the S&P 500 index and two proxy variables corresponding to production level (number of employees and operating costs).

The data referring to each company in the S&P 500's turnover during 2000–2009 were extracted from the Thomson Reuters database. However, the data for the proxy variables of the production level were available by sector from the North American Industry Classification System (NAICS).

Thus, the analyzed companies were grouped by NAICS sector, as shown in the Supplementary Material Table 1. We used 17 sectors with the following names and corresponding NAICS codes: Agriculture, Forestry, Fishing and Hunting (11), Mining (21), Utilities (22), Construction (23), Manufacturing (31–33), Wholesale Trade (42), Retail Trade (44–45), Transportation and Warehousing (48-49), Information (51), Finance and Insurance (52), Real Estate Rental and Leasing (53), Professional, Scientific and Technical Services (54), Administrative and Support and Waste Management and Remediation Services (56), Educational Services (61), Health Care and Social Assistance (62), Art, Entertainment and Recreation (71), and Accommodation and Food Services (72). Given its residual character, we ruled out the Business Administration (55) and Other Services (81) sectors. Similarly, to be more precise in our proposed classifications, we used the Bloomberg website, where one can enter each listed company's acronym to obtain additional information.

With respect to the sectors presented in the Supplementary Material Table 1, the category of Utilities includes activities that consist of the supply of gas and electricity generated using energy sources such as nuclear, solar, wind, hydraulic, geothermal, biomass, etc. The Manufacturing sector includes the largest number of companies and a diverse array of activities, including production machinery, textiles, food, drinks, tobacco, chemicals, wood, etc.

After performing the NAICS sector classification, we see that the quarterly data referring to operating costs, although they fit with the classification, are further aggregated. Therefore, to make homogeneous estimations that incorporate the independence of the estimation alternative used (e.g., number of employees or operating costs), we modified the sectors proposed in the NAICS classification.

Consequently, Agriculture, Forestry, Fishing and Hunting, and Mining were combined into a single sector. The same was done with Finance and Insurance, and Real Estate Rental and Leasing, Professional, Scientific and Technical Services, and Administrative and Support, and Waste Management and Remediation Services, Educational Services and Health Care and Social Assistance and finally, Arts, Entertainment, and Recreation and Accommodation and Food Services.

Following that modification, we had 12 activity sectors to consider (see Table 1).

**Table 1:** **Adapted sectoral NAICS classification used in this study**.

**No**	**Analyzed activity sectors**
S1	Leisure and accommodation
S2	Health care and educational services
S3	Wholesale trade
S4	Retail trade
S5	Construction
S6	Forest and mining exploitation
S7	Finance and real estate
S8	Information
S9	Manufacturing
S10	Professional and administrative services
S11	Transportation and warehousing
S12	Utilities

Source: Own elaboration based on http://www.naics.com/search.htm.

Once we defined our sectors, we followed the same methodology that we used for the inflation rate and applied year-to-year calculations to the rest of the variables to avoid seasonality problems.

In addition, we conducted different tests to analyze the seasonality of the series: Augmented Dickey-Fuller (ADF), Phillips-Perron (PP), and Kwiatkowski-Phillips-Schmidt-Shin (KPSS). The results of the tests are not shown, but they are available upon request.

The “turnover” variable exhibits a unit root in levels, although the variable in the first differences was stationary at standard levels of significance.

#### Proxy variable of production level

As previously stated, because the S&P 500-listed companies' production volume is a variable that cannot be observed directly, we were forced to seek a proxy variable. One important contribution of this study is the use of the proxy variable of production level, not only the number of employees (Jareño, 2005; Jareño and Navarro, 2010) but also some alternative variable such as operating costs. However, for comparison purposes, we performed the estimation of the FT coefficients considering the two variables selected as proxies for production level.

#### A. operating costs: companies of the S&P 500

The data referring to operating costs were obtained on a monthly basis from the website of the *Bureau of Labor Statistics* in the form of index numbers for the year 2002. These costs are representative of production value and were extracted from public reports published annually by the actual companies studied. In this way, we extracted the information corresponding to the 12 sectors included in Table 1 for 2000–2009.

To homogenize the data, we obtained the desired frequency by determining quarterly means and then doing year-to-year calculations. The series of operating costs is stationary in its first differences according to the tests noted above.

#### B. number of employees: S&P 500 companies

The other alternative that we considered as a proxy variable for the production level of S&P 500 companies, following Jareño (2005) and Jareño and Navarro (2010), is number of employees. The reason that we selected this variable is that it has a direct and positive relationship with production volume. Accordingly, we were able to compare the results obtained using both variables and evaluate them for consistency.

Thus, data related to the number of employees was extracted from the website of the *Bureau of Labor Statistics*, on a monthly basis, for the 17 activity sectors included in the Supplementary Material Table 1 (in thousands of employees and for the sample period). However, we were required to adapt this information by adding the data in the 12 above-described sectors and by obtaining quarterly and annual frequencies.

The stationarity and unit-root tests confirm the stationarity of the time series in first differences.

Finally, the Supplementary Material Table 2 collects the primary descriptive statistics of the variables used in this study. We observe that the mean and median are positive for all of the sectors using the variables “turnover” and “operating costs” and the variable “inflation,” which indicates growth for both variables during the studied period. On the contrary, the mean and median of the variable “number of employees” shows a negative sign for more than half of the sectors analyzed, which indicates a reduction. With respect to standard deviation, the variable “turnover” is recurrently more volatile than the proxy variables for production level (“operating costs” and “number of employees”) and “rate of inflation,” fundamentally S6 and S1. The asymmetry exhibited by the explanatory variables is clearly negative, whereas the dependent variable (“turnover”) does not show a clear sign. All of the variables exhibit excessive kurtosis.

After studying the data, we estimated the FT coefficients according to the proposed sector classifications. The idea was to analyze companies' ability to transmit inflation shocks to their prices, noting that those inflation shocks are a function of the sector of economic activity in which the companies were classified during 2000–2009; that analysis is this study's primary objective. In addition, we used two alternative estimates: the first using operating costs and the second using number of employees.

### US sectoral results of FT coefficients using an alternative estimation of US FTC

Starting with Equations (7) and (11) derived from previous studies, we created a system of 12 equations (one for each activity sector analyzed) using the following format:

(15) $$\begin{array}{rcl} d\left(\frac{{CNeg}_{ti}-{CNeg}_{t-4i}}{{CNeg}_{t-4i}}\right) & = & \beta_{0}+\beta_{1}\cdot d\left(\frac{{CteOp}_{ti}-{CteOp}_{t-4i}}{{CteOp}_{t-4i}}\right)+\\ \beta_{2}\cdot d\left(\frac{{TInf}_{t}-{TInf}_{t-4}}{{TInf}_{t-4}}\right)+\varepsilon_{t} \end{array}$$

where *CNeg*_*ti*_ refers to the turnover for each sector *i, CteOp*_*ti*_ reflects the operating costs of the different sectors *i* and *Tinf*_*t*_ the American inflation rate for 2000–2009. In addition, β_0_ represents the independent term, β_1_ is the coefficient that measures the variation in turnover for each activity sector as a result of unit variations in operating costs, β_2_ is the FT coefficient—that is, it measures the capability of companies in the sector to transmit to prices (and, therefore, to turnover) inflation shocks in the economy—and ε_*t*_ alludes to the error term.

This study used the Seemingly Unrelated Regression (SUR) method to obtain the FT coefficients. This method avoided problems related to heteroscedasticity and a possible contemporary correlation between the different equations' error terms (i.e., autocorrelation). The results can be seen in Table 2.

**Table 2:** **Estimation of FT coefficients with “operating costs” variable**.

**Sectors**	**T. Independent β_0_**	**Operating Costs β_1_**	**Inflation Rate β_2_**	***R*^2^**	**Adjusted *R*^2^**
S1	0.0230 (0.4669)	9.2259 (3.1440^b^)	2.7595 (0.7215)	0.1760	0.1315
S2	–0.0006 (–0.0130)	5.0357 (1.4579^a^)	–0.0025 (–0.0007)	0.0284	−0.0240
S3	–0.0085 (–0.6147)	0.0900 (0.4134)	1.3870 (1.3515)	0.0298	−0.0225
S4	–0.0047 (–0.7151)	1.3441 (2.1993^b^)	–0.6372 (–1.2141)	0.1207	0.0731
S5	–0.0099 (–0.6132)	0.2153 (0.4182)	0.6797 (0.5515)	0.0123	−0.0410
S6	–0.0323 (–0.2820)	0.1563 (0.1035)	7.9961 (0.9299)	0.0227	−0.0300
S7	–0.0091 (–0.4076)	–0.6543 (–0.4972)	4.2943 (2.5848^b^)	0.1339	0.0871
S8	0.0022 (0.2554)	1.2861 (2.5949^b^)	–0.6974 (–1.0272)	0.1205	0.0729
S9	–0.0004 (–0.0720)	0.9326 (4.2413^b^)	3.5957 (7.0050^b^)	0.7138	0.6984
S10	0.0004 (0.0271)	1.8979 (3.0379^b^)	0.2653 (0.2009)	0.0483	−0.0030
S11	–0.0044 (–0.2434)	0.0142 (0.0211)	2.8149 (1.9846^b^)	0.0975	0.0488
S12	0.0015 (0.0375)	1.9491 (2.3242)	4.3839 (1.4127^a^)	0.0983	0.0495

This table shows the results of the model proposed by Jareño and Navarro (2010) to estimate the FT capability of companies, but applying the alternative proxy variable representing the production level. The sample extended from 2000–2009 and the regression was estimated using SUR methodology:

$$\begin{array}{c} d\left(\frac{{CNeg}_{ti}-{CNeg}_{t-4i}}{{CNeg}_{t-4i}}\right)=\beta_{0}+\beta_{1}\cdot d\left(\frac{{CteOp}_{ti}-{CteOp}_{t-4i}}{{CteOp}_{t-4i}}\right)+\beta_{2}\cdot d\left(\frac{{TInf}_{t}-{TInf}_{t-4}}{{TInf}_{t-4}}\right)+\varepsilon_{t} \end{array}$$

where Cneg_ti_ refers to the turnover for each sector i, CteOp_ti_ reflects the operating costs of the different sectors i, TInf_t_ reflects the American inflation rate and ε_t_ reflects the error term.

a p < 0.15;

b p < 0.05 (t-statistics in parenthesis).

Table 2 shows the estimated coefficients β_0_, β_1_, and β_2_, which represent the FT coefficients. As seen, in four sectors (S7, S9, S11, and S12), the results are significantly different from zero and the sign of the FT coefficient is positive: “Finance and Real Estate,” “Manufacturing,” “Transportation and Warehousing,” and “Utilities.” For the rest of the sectors, the results are not significantly different from zero.

### US sectoral results of FT coefficients using the Jareño and Navarro (2010) methodology

Next, we estimated the FT coefficients following the method proposed by Jareño and Navarro (2010) and incorporating as the proxy variable for production level the aggregated number of employees by sector. In this way, we were able to compare the results obtained using both procedures to analyze their robustness.

Some previous studies have applied the Jareño and Navarro (2010) methodology. Thus, the robustness of this proposal has been tested, at international level, mainly in Peiró (2016), and, at Spanish level, in Ballester et al. (2011), Jareño and Tolentino (2012), and Díaz and Jareño (2013), among others. Moreover, this and other previous research has relied on this FT methodology in order to include a better explanation of each evidence found.

Therefore, the model used for the estimation is shown in the following equation:

(16) $$\begin{array}{rcl} d\left(\frac{{CNeg}_{ti}-{CNeg}_{t-4i}}{{CNeg}_{t-4i}}\right) & = & \beta_{0}+\beta_{1}\cdot d\left(\frac{{NEmp}_{ti}-{NEmp}_{t-4i}}{{NEmp}_{t-4i}}\right)+\\ \beta_{2}\cdot d\left(\frac{{TInf}_{t}-{TInf}_{t-4}}{{TInf}_{t-4}}\right)+\varepsilon_{t} \end{array}$$

where *Nemp*_*ti*_ reflects the number of employees in each activity sector *i* during 2000–2009. In addition, β_0_ represents the independent term, β_1_ is the coefficient that measures the variation in turnover in each activity sector resulting from a unit variation in their number of employees, β_2_ is the FT coefficient, and ε_*t*_ is the error term.

Using the SUR methodology, we estimated a system consisting of 12 equations (one per sector). The results can be observed in Table 3 as shown below.

**Table 3:** **Estimation of FT coefficients with the variable “no of employees”**.

**Sectors**	**T. independent β_0_**	**No of employees β_1_**	**Inflation rate β_2_**	***R*^2^**	**Adjusted *R*^2^**
S1	0.0223 (0.4285)	4.2656 (1.8030^b^)	4.1586 (1.0392)	0.0943	0.0453
S2	0.0027 (0.0592)	2.7374 (0.9074)	0.6956 (0.1970)	0.0199	–0.0330
S3	–0.0058 (–0.4162)	1.9704 (1.0556)	0.9028 (0.8147)	0.0563	0.0053
S4	–0.0023 (–0.3491)	3.3184 (3.4818^c^)	–1.0549 (–1.9620^c^)	0.1588	0.1134
S5	0.0030 (0.1819)	2.5585 (2.2323^c^)	–0.3211 (–0.2555)	0.0840	0.0345
S6	–0.0526 (–0.4647)	–3.9121 (–2.1227^c^)	11.2182 (1.3139)	0.0530	0.0018
S7	–0.0025 (–0.1089)	1.5921 (0.7775)	3.6408 (2.0031^c^)	0.1502	0.1042
S8	0.0029 (0.3324)	1.7927 (2.2067^c^)	–0.5894 (–0.8547)	0.0834	0.0338
S9	–0.0020 (–0.2988)	0.1272 (0.2435)	4.4087 (7.6214^c^)	0.6515	0.6327
S10	–0.0038 (–0.2222)	0.4177 (0.6564)	0.6875 (0.4762)	0.0448	–0.0067
S11	–0.0030 (–0.1691)	0.7492 (0.6709)	2.5387 (1.7946^c^)	0.1059	0.0576
S12	0.0004 (0.0110)	–3.3395 (–1.1030)	4.5480 (1.4383^a^)	0.0670	0.0166

This table gathers the results of the model proposed by Jareño and Navarro (2010) to estimate the FT capability of companies, using the number of employees as proxy variable for production level. The sample extended from 2000–2009 and the regression was estimated using the SUR methodology:

$$\begin{array}{l} d\left(\frac{{CNeg}_{ti}-{CNeg}_{t-4i}}{{CNeg}_{t-4i}}\right)=\;\beta_{0}+\beta_{1}\cdot d\left(\frac{{NEmp}_{ti}-{NEmp}_{t-4i}}{{NEmp}_{t-4i}}\right)+\beta_{2}\cdot d\left(\frac{{TInf}_{t}-{TInf}_{t-4}}{{TInf}_{t-4}}\right)+\varepsilon_{t} \end{array}$$

where Nemp_ti_ reflects the number of employees of each activity sector i, Cneg_ti_ reflects the turnover for each sector i, TInf_t_ reflects the American inflation rate and ε_t_ reflects the error term.

a p < 0.15;

b p < 0.10; ^c^p < 0.05 (t-statistics in parenthesis).

According to this alternative estimation, we see that in addition to the four sectors that present a significantly positive FT ability in the previous estimation, S4 “Retail Trade” shows results that are significantly different from zero even though in this case, the sign of the FT coefficient is negative.

In general, the results are very consistent with the use of one or the other proxy variable for production level.

Adjustment of the model is very high in the sectors that exhibit a significant FT ability, reaching almost 70% in S9 with the use of operating costs to approximate production level.

#### Interpretation of sectoral results

Following the logic of Asikoglu and Ercan (1992) and Jareño and Navarro (2010), there are clear differences between the FT coefficients obtained for the industries analyzed. This demonstrates that not all industries have the same ability to maintain growth in their turnover and/or profits during an inflationary period, given that multiple differential factors can affect certain industries: market power, competition level, competitive strategies, economic context, etc.

In general, it is true for both estimation formulas that the significant FT coefficients have a positive sign. This leads to the conclusion that in the face of increased inflation, turnover also exhibits a positive variation because companies are capable of transmitting—to a lesser or greater degree—inflation shock to the price of their products or services. That is, the sign of the FT coefficients indicates a positive relationship between both variables for the sectors for which data are significant (S7 Finance and Real Estate, S9 Manufacturing, S11 Transportation and Warehousing and S12 Utilities).

On the other hand, S4 Retail Trade is only significant when using the second alternative (using the number of employees as the proxy variable for production level) and holds a negative sign. This confirms that inflation shocks have a negative effect on that sector's profits.

#### S7 financial and real estate sector

The FT coefficients estimated using both alternative procedures are 4.2943 (using operating costs as a proxy variable for production level) and 3.6408 (using number of employees). The coefficients obtained are similar. Therefore, both estimation procedures could be substituted for each other, which in principle, verifies our selection of operating costs as an alternative variable to number of employees.

From an economic perspective, we must highlight that the sectors related to real estate and finance suffered greatly from the 2007 financial crisis. However, until then, the financial sector, and to a greater degree the real estate sector, was marked by a continuous increase in the price of its services provided and products (i.e., homes sold/rented). This could explain the extremely high value of its FT coefficient.

However, price increases ended with the burst of the real estate bubble, which presumably decreased those sectors' ability to transmit inflation shocks to their prices. Therefore, it would be interesting to perform estimations of the FT capability differentiated by sub-periods as a function of the economic cycle, but the scarcity of data prevented us from doing so.

#### S9 manufacturing sector

The FT coefficients estimated using both procedures are 3.5957 (using operating costs) and 4.4087 (using the number of employees). Again, the value of the coefficients is similar and significant in both cases, confirming the consistency of the results obtained.

This sector includes manufacturing related to machinery for production, textiles, food, beverages, tobacco, chemicals, wood, etc. Therefore, in the majority of cases, the companies that operate in this sector have great market power. For example, large American tobacco companies enjoy an oligopolistic position. A similar phenomenon occurs, to a lesser degree, with the production of certain production machinery, because the United States is not only at the vanguard of new technology but also makes significant investments into research, development and innovation. This could explain the elevated values of the FT coefficients, which are equivalent to those of the financial and real estate sector.

#### S11 transportation and warehousing sector

The FT coefficients estimated are 2.8149 and 2.5387, respectively, for the use of operating costs and the number of employees as proxy variables for production level. It is seen again that the values obtained are practically the same.

In addition, the coefficients are lower than those of the sectors analyzed previously: the transportation and warehousing sector has no companies that enjoy a clearly dominant position in the market because it is a sector with an elevated level of competition (even foreign). However, the United States is the biggest world power, which makes the country brand a marketing tool with great international reach and therefore, the demand for American products is overwhelming. Thus, orders of American products must be transported to their destinations and, until their departure, they are stored in the place of origin. This could explain the values obtained for the coefficients.

#### S12 utilities

Utilities constitute the last of the four sectors for which the FT coefficients are significant using both estimation procedures. The values obtained are 4.3839 and 4.5480, respectively, for the use of each proxy variable for production level. The values taken by the coefficients are similar, as is the level of significance.

This sector includes activities consistent with the supply of gas and electricity generated through energy sources such as nuclear, solar, wind, hydraulic, geothermal, biomass, etc., some of which are subject to government regulation. Therefore, these businesses are regulated. Consequently, if the economy experiences an inflation shock, then US authorities can exert their control over these companies, transmitting inflation to prices. This justifies the extremely elevated FT coefficients resulting from this estimation.

#### S4 retail trade sector

As mentioned previously, the coefficients relative to this sector are only significant when they are estimated using the proxy variable “number of employees” (not when using operating costs).

In addition, their sign is negative (–1.0549), indicating that in the face of an increase in inflation, the turnover of the companies included in this sector decreases. There are several possible economic explanations for this result:

1. On the one hand, even though inflation shock can be transmitted to the prices of products and/or services, number of sales can experience such a reduction that in the end, turnover goes down.
2. On the other hand, companies in this sector can lobby for a price freeze on their products, so that although their sales and income remain the same, other operating costs may go up, thus causing turnover to go down.
3. Finally, it may be that this coefficient shows that companies in this sector lack the ability or know-how to transmit inflation shocks to prices.

The remaining unmentioned sectors (S1, S2, S3, S5, S6, S8, and S10) exhibit FT coefficients that are not significantly different from zero. However, there are subtleties worth mentioning with respect to those cases for which the FT coefficients take on a different sign using one estimation procedure from that when using the other estimation procedure.

- First, S5 Construction is one of the sectors in which we see this contradiction; however, we must recall that the coefficients are not significant and that this sector experienced a convoluted situation during the first decade of this century.

Until the middle or final months of 2007, the number of homes grew exponentially, driven by the real estate bubble that formed during those years. Many investors bought and sold homes with the single goal of obtaining profits, without stopping to think whether the sale value represented the home's real value. This created a difficult future for the real estate sector and by extension, for the construction sector, which did later arrive. Construction has been one of the most affected sectors from the beginning of the global economic crisis, forcing the closing of many construction businesses that had been encouraged by artificial optimism.

- Second, the other sector in which the signs of the coefficients were not the same using both estimation alternatives is S2 Health Care and Educational Services. However, in this case, we can outline the FT coefficients to obtain improved specificity by subsector— Health Care and Social Assistance and Educational Services—even though this is only possible when using number of employees. It is important to remember that the reason for using 12 sectors, not the 17 sectors proposed in the NAICS classification, because operating costs were not available at that level of differentiation.

We did the same for those sectors that can be broken down into two activity sub-sectors, such as S1 Leisure and Accommodation, S6 Forest and Mining Exploitation, S7 Finances and Real Estate, and finally, S10 Professional and Business Services. In this way, we propose a new system with 10 equations, estimated using SUR methodology through Equation (16), although it was only applied to the sub-sectors to which we have alluded because we can differentiate an FT coefficient for each of them. The results are shown in Table 4.

**Table 4:** **Estimation of FT coefficients with the variable “no of employees” for the differentiated sub-sectors**.

**Sectors**	**T. independent β_0_**	**No of employees β_1_**	**Inflation rate β_2_**	***R*^2^**	**Adjusted *R*^2^**
Health Care	–0.0025 (–0.0820)	–8.0708 (–0.7639)	1.0205 (0.4385)	0.0080	–0.0455
Educational Services	0.0031 (0.2207)	–0.7165 (–0.5056)	0.1272 (0.1191)	–0.011	–0.0665
Leisure	0.0201 (0.4202)	3.8708 (1.2134)	3.9600 (1.0782)	0.0928	0.0438
Accommodation	–0.0014 (–0.3543)	1.9517 (3.1047^b^)	0.8274 (2.5132^b^)	0.3619	0.3274
Forest Exploitation	–0.0163 (–0.2632)	–1.4935 (–0.6198)	–3.2681 (–0.703)	0.0351	–0.017
Mining	–0.0215 (–0.2853)	–0.1242 (–0.446)	11.812 (2.0304^b^)	0.0989	0.0502
Finance	0.0029 (0.2786)	2.9888 (1.7547^a^)	1.3798 (1.7229^a^)	0.1641	0.1189
Real Estate	–0.0133 (–0.6791)	–3.2380 (–1.089)	3.4235 (2.1243^b^)	0.0762	0.0262
Professional Services	0.0061 (0.0096)	1.2795 (1.9771^b^)	0.1294 (0.2546)	0.1386	0.0921
Administrative Services	0.0017 (0.1820)	1.2876 (2.0129^b^)	–0.2197 (–0.269)	0.0805	0.0308

This table shows the results of the model proposed by Jareño and Navarro (2010) to estimate the FT capability of companies, applying the number of employees as the proxy variable for production level. The sample extended from 2000–2009 and the regression was estimated using the SUR methodology:

$$\begin{array}{l} d\left(\frac{{CNeg}_{ti}-{CNeg}_{t-4i}}{{CNeg}_{t-4i}}\right)=\beta_{0}+\beta_{1}\cdot d\left(\frac{{NEmp}_{ti}-{NEmp}_{t-4i}}{{NEmp}_{t-4i}}\right)+\beta_{2}\cdot d\left(\frac{{TInf}_{t}-{TInf}_{t-4}}{{TInf}_{t-4}}\right)+\varepsilon_{t} \end{array}$$

where Nemp_ti_ reflects the number of employees in each activity sector i, Cneg_ti_ reflects the turnover for each sector i, TInf_t_ reflects the American inflation rate and ε_t_ reflects the error term.

a p < 0.10;

b p < 0.05 (t-statistics in parenthesis).

Before carrying out the disaggregated estimate of the FT coefficients, sector S2 Health Care and Educational Services showed a negative sign in the case of alternative 1 (operating costs) and a positive sign in the case of alternative 2 (number of employees). After breaking down the calculations for the different sub-sectors, one can see that the signs for both continue to be positive through the second alternative and are not significant.

Similarly, the FT coefficients of S7 Finance and the Real Estate Sector, which were positive and significant in the previous analysis, maintain their sign and level of significance when broken down into two sub-sectors.

Sub-sectors with significant coefficients resulting from the separate analysis include Accommodation Services (included in S1 of our NAICS adapted classification) and Mining (within S6). The FT coefficient for accommodation services (0.8274) is relatively low, given that it is a sector with a great deal of competition. However, we must remember that it is a positive and significant coefficient, which suggests that businesses in the sector are capable of partially transmitting inflation shocks to service prices. Conversely, Mining shows a very elevated value (11.812), which could be due to the sector's need to use natural resources that are increasingly scarce and expensive. This would explain the elevated ability to absorb inflation exhibited by the companies in this sector, given that an increase in the price of those resources would not disincentivize their purchase by clients with great purchasing power, given that they could be considered “luxury” goods (gold, silver, etc.).

## The relationship between FT coefficients and stock prices

This section addresses the study of the relationship between estimated sectoral FT coefficients and variations in stock prices corresponding S&P 500 companies during 2000–2009.

In that sense, we have data for the FT coefficients by sector, notwithstanding the fact that because we made estimations using two alternatives, we have sectoral data derived from each of them. In this case, we consider a simple cross-section regression that offers important information.

Conversely, for the case of the stock prices of S&P 500 companies, we have quarterly data for 2000–2009 (40 observations), extracted from the Thomson Reuters database. This means that we aggregated stock trading data from companies in the 12 sectors listed in the NAICS classification, as adapted for this study. We then calculated the variation in stock prices during the sample period to analyze their relationship with the previously estimated FT coefficients:

(17) $$\begin{array}{l} {△Cot}_{i}=\frac{{Cot}_{4T\;2009i}-{Cot}_{1T\;2000i}}{{Cot}_{1T\;2000i}} \end{array}$$

where *Cot*_*i*_ refers to the quarterly prices of stocks of companies included in sector *i* and *T* represents the quarter in question.

Following the work of Asikoglu and Ercan (1992), we estimated, using ordinary least squares (OLS) adjusted by White (to avoid heteroscedasticity problems), Equation (18), which relates the variation in quarterly stock prices to the estimated FT coefficients (both at the sector level). Our goal was to analyze the value and sign of the coefficient that relates to both magnitudes and statistical significance. The equation is as follows:

(18) $$\begin{array}{l} {△Cot}_{i}=\delta_{0}+\delta_{1}\cdot {CFT}_{i} \end{array}$$

where *CFT*_*i*_ gathers the estimated FT coefficients for each sector *i*, δ_0_ is the independent term, and δ_1_ is the coefficient that relates the variations in stock prices with the FT coefficients.

**Figure 1:**
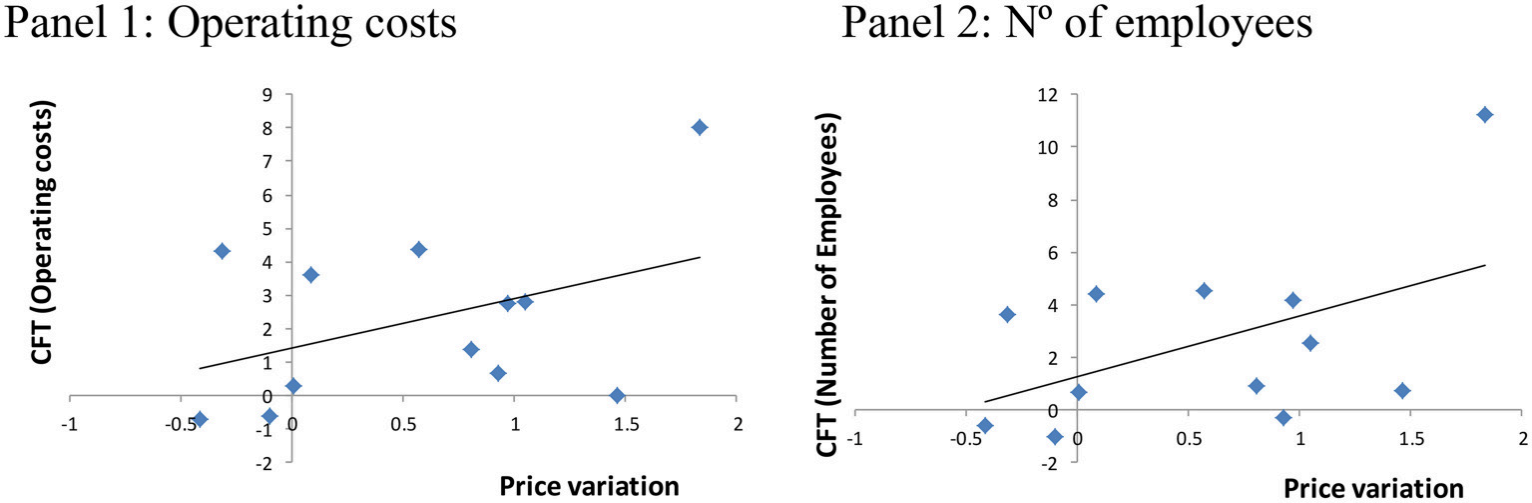
**Relationship between FT capability and variation in stock prices at the sector level**.

As indicated previously, this equation was estimated twice (Table 5 and Figure 1): once with the FT coefficients obtained through the first alternative (operating costs) and second, with the coefficients resulting from the second alternative (number of employees). Thus, we were able to compare the results of both alternatives and to verify (or not) the positive relationship between stock prices and the FT coefficients, as defended by Asikoglu and Ercan (1992) and Jareño and Navarro (2010).

**Table 5:** **Estimation of the relationship between sectoral FT coefficients and variations in stock prices**.

	**T. independent δ_0_**	**CFT δ_1_**	***R*^2^**	**Adjusted *R*^2^**
**PANEL 1: OPERATING COSTS**				
*ΔCot*	0.3154 (1.1727)	0.1152 (1.3927)	0.1705	0.0875
**PANEL 2: NO OF EMPLOYEES**				
*ΔCot*	0.3059 (1.2568)	0.1040 (2.3261^a^)	0.2404	0.1644

This table gathers the results of the model proposed by Asikoglu and Ercan (1992) to study the relationship between the FT ability of companies classified at the sector level and changes in their stock prices. The sample extends from 2000–2009 and the regression was estimated by ordinary least squares (OLS) adjusted by White (to avoid heteroscedasticity issues):

$$\begin{array}{l} {△Cot}_{i}=\delta_{0}+\delta_{1}\cdot {CFT}_{i} \end{array}$$

where CFT_i_ represents the FT coefficients estimated for each sector i, δ_0_ represents the independent term and δ_1_ represents the coefficient that relates variations in stock price with FT coefficients.

a p < 0.05 (t-statistics in parenthesis).

As shown above, the δ_0_ coefficient is not significant if we use operating costs as the proxy for production level. Therefore, although it has a positive sign, as argued by Asikoglu and Ercan (1992) and Jareño and Navarro (2010), it does not have statistical significance. However, when estimating the model proposed with FT coefficients resulting from using the number of employees as the proxy variable for production level, the δ_1_ coefficient (= 0.1040) is similar in magnitude to the previous estimation, but in this case it is significant and has a positive sign. In this way, we see results in line with the existing literature.

According to Asikoglu and Ercan (1992), Jareño and Navarro (2010) and our own results, there is a positive relationship between changes in stock prices and FT capability, which varies as a function of the sector under consideration. As a result, investors are willing to pay a higher price for stock in those companies that are capable of transmitting larger portions of inflation shocks to their product prices, which would mean, in the end, a growth in profits/dividends. Accordingly, the empirical evidence presented suggests that FT ability has an effect on stock prices.

Similarly, Asikoglu and Ercan (1992) argue for a negative relationship between inflation and stock prices at the sector level in the US and therefore, the same is true for the relationship between inflation and FT capability. Following this logic, increasing the FT capability is associated with higher stock prices and in industries in which such coefficients are higher, stock prices are less sensitive to inflation shocks. This last conclusion is demonstrated for our case in Table 6, which corroborates the primary idea of the FT model proposed by Asikoglu and Ercan (1992): when inflation increases, pressure on stock prices through the discount rate is counteracted to a certain point by increments in the growth expected of the nominal rate of return of equity securities. This compensatory effect is positively related to the FT coefficient of the industry in question.

**Table 6:** **Relationship between stock price variation and FT coefficients at the sector level**.

**Sectors**	**Stock price mar-00**	**Stock price dec-09**	**Var. stock price 2000/09**	**CFT (O. costs)**	**CFT (No of employees)**
S1	134.66	265.36	0.9705	2.7596	4.1586
S2	159.23	392.18	1.4629	−0.0026	0.6957
S3	208.50	376.45	0.8056	1.3871	0.9028
S4	873.38	1221.52	0.3986	−0.6373	−1.0549
S5	191.99	369.76	0.9259	0.6797	−0.3212
S6	337.70	957.40	1.8350	7.9962	11.2183
S7	3901.04	2675.71	−0.3141	4.2943	3.6408
S8	2153.39	1265.73	−0.4122	−0.6975	−0.5895
S9	7850.04	7198.34	−0.0830	3.5958	4.4087
S10	511.91	503.34	−0.0167	0.2653	0.6876
S11	257.50	527.28	1.0477	2.8149	2.5388
S12	722.07	1134.29	0.5709	4.3840	4.5480

CFT collects the estimated FT coefficients for each sector.

Comparing the column “Var. Stock Price 2000/09,” which indicates the variation in stock price during 2000–2009 with the FT coefficients (last two columns), we see that in general, in those sectors in which these coefficients are higher, variation is lower. This fact can be verified for S9 Manufacturing and S7 Finance and Real Estate. However, it does not hold true for S6 Forest and Mining Exploitation because it has the highest FT coefficient using both estimation alternatives, but the variation in the stock prices is not the lowest at the sector level, although it does show one of the highest stock prices.

In contrast, empirical evidence presented by Asikoglu and Ercan (1992) suggests that in addition to the effect of FT ability on stock prices, the coefficients that measure it in different sectors differ significantly. Therefore, we analyzed whether different sectors' different capacities to absorb inflation (analyzed in a pair-wise fashion) exhibit statistical significance.

To that end, we performed the Wald test for both estimation alternatives (see Table 7). That test is a symmetrical matrix that evaluates, in a pair-wise fashion, whether the estimated FT coefficients are significantly different. There is no reason to estimate the principal diagonal because it compares of each sector to itself (with an equality probability of 100%), and the upper diagonal exactly coincides with the lower one.

**Table 7:** **Wald test to evaluate whether sectoral FT coefficients are pair-wise significantly different (two alternatives)**.

	**1^1^**	**2**	**3**	**4**	**5**	**6**	**7**	**8**	**9**	**10**	**11**	**12**
**OPER. COSTS**												
S1	−											
S2	0.3335	−										
S3	0.1206	0.1549	−									
S4	0.7945	0.0336	2.545(a)	−								
S5	0.2434	0.0363	0.1698	1.3089	−							
S6	0.325	0.9589	0.6191	0.9987	0.7409	−						
S7	0.1576	1.1572	2.605(a)	7.589(c)	2.493(a)	0.1807	−					
S8	0.7593	0.0371	3.249(b)	0.0044	1.014	1.0126	9.148(c)	−				
S9	0.0506	1.046	3.474(b)	37.525(c)	4.851(c)	0.2587	0.1698	24.899(c)	−			
S10	0.4637	0.004	0.3374	0.4249	0.0614	0.7496	4.089(c)	0.4264	9.063(c)	−		
S11	0.0002	0.5851	0.6892	5.563(c)	1.4073	0.4062	0.5814	5.1892(c)	0.282(a)	2.5233	−	
S12	0.1578	1.2945	0.9851	2.482(a)	1.2179	0.3112	0.0008	2.6088(a)	0.0620	1.4793	0.4216	−
**NO. OF EMPLOYEES**												
S1	−											
S2	0.5062	−										
S3	0.5994	0.0035	−									
S4	1.6694	0.2591	2.0216	−								
S5	1.0114	0.0814	0.4496	0.3731	−							
S6	0.5382	1.5937	1.5445	2.0567	1.8831	−						
S7	0.0163	0.5448	1.7531	5.469(c)	2.566(a)	0.7619	−					
S8	1.3257	0.1322	1.5906	0.2525	0.0363	1.9183	5.283(c)	−				
S9	0.0041	1.118	7.182(c)	50.85(c)	12.873(a)	0.6165	0.1753	31.973(c)	−			
S10	0.7879	0.0003	0.0105	1.4614	0.3398	1.4094	1.7796	0.6507	8.9729(c)	−		
S11	0.1737	0.2573	0.8524	5.839(c)	2.538(a)	1.1796	0.2699	4.017(c)	1.5178	1.1544	−	
S12	0.0075	0.9202	1.3913	2.937(b)	2.106(a)	1.0583	0.0743	2.628(a)	0.0017	1.2259	0.7399	−

Table 7 shows the results of the test performed, which offers the value of the Chi-squared distribution for each pair of FT coefficients and for the level of significance. Accordingly, if two coefficients are significantly different, then the ability of companies in one sector to absorb inflation is significantly different then the ability exhibited by companies in the other sector.

As expected, notably different results are obtained depending on which sectors we compared. We must highlight that in all cases in which the pairs of FT coefficients are significantly different (for both of the estimations been proposed here), there is involvement by one of the sectors for which the estimated coefficients are shown to be significant in Tables 3, 4.

The remaining sectors, analyzed in a pair-wise fashion, offer results that are not significant, which is why we can assume that the capability of companies in one sector to absorb inflation is significantly different from that of companies in an alternate sector that is being compared.

The fact that we have found cases in which the differences are significant and others in which they are not corroborates the idea that sectors have different abilities to transmit inflation shocks to the price of their products and services. Therefore, our results are in line with the studies carried out by Asikoglu and Ercan (1992), Jareño (2005), and Jareño and Navarro (2010).

## Overall results

In short, estimates about the capability of American companies to transmit inflation shocks to the prices of the products that they sell and/or the services that they provide (i.e., estimates about the FT capability) seem to be quite different among sectors. These results are in line with previous literature, such as Asikoglu and Ercan (1992), Jareño (2005), Ertek (2009), and Jareño and Navarro (2010), and, finally, Ang et al. (2011), at company level.

Second, there may be a positive relationship between changes in stock prices and FT capability, and also, this relationship varies among sectors. Previous studies (Asikoglu and Ercan, 1992; Ertek, 2009; Díaz and Jareño, 2013; Taylor, 2013) find similar evidence.

Finally, as suggested by Asikoglu and Ercan (1992) and Jareño and Navarro (2010), the FT coefficients by sector are statistically and significantly different, and our study corroborate it. As a result, this research may confirm that investors should consider the FT capability in decision-making (Kusev and van Schaik, 2011).

## Discussion

This research is based on the impression that the investor behavior may be different depending on the company's capability to transmit inflation shocks to the prices of its products and services (i.e., the FTC), since investors seek protection from interest and inflation rate changes, among other sorts of risk. Additionally, higher FT capability is associated with higher stock prices, and in industries in which FT coefficients are higher, stock prices are less sensitive to inflation shocks. Therefore, the FT capability is a key factor in investment decisions.

To that end, the primary objective of this study is to evaluate the ability of American companies (listed in the S&P 500 index) to transmit inflation shocks to the prices of their products and/or services, grouping companies into 12 sectors based on their activity, according to the adapted North American NAICS classification that we propose. The study period is inclusive of 2000–2009 and the data frequency is quarterly, which also represents an improvement compared to previous studies with semiannual frequencies.

First, we made two alternate estimations of companies' power to transfer inflation shocks to their prices as a function of two proxy variables for production level (a variable that is not directly observable): operating costs and number of employees. The estimated FT coefficients differ considerably as a function of each sector, in line with previous studies (Asikoglu and Ercan, 1992, and Jareño and Navarro, 2010). This result can be explained as a function of the peculiarities that affect companies in each sector differently, such as level of competition, competitiveness strategies, market share, economic context, etc. Moreover, this result would explain differences in decision-making by investors depending on the FT capability.

This research also demonstrates the existence of a positive relationship between the variation exhibited by stock prices in the companies of a single sector and its corresponding FT coefficient. This positive relationship exists because investors are willing to pay a higher price for stocks when a larger part of the inflation rate provided is transmitted to them in the form of growth in earnings/dividends obtained. Thus, increments in FT coefficients are associated with higher stock prices.

Similarly, the empirical evidence indicates that in those industries that have a greater ability to transmit to prices, in the majority of cases, stock prices are less sensitive to inflation shocks. This is not due to the presence of a negative relationship between inflation and stock price levels, although it is true that in those sectors in which FT capability is relatively high, inflation shocks are transmitted, practically in their entirety, to the price of products sold and services provided. Therefore, investors trust stock prices given that their valuation can remain intact.

In general, we see that the results obtained are in agreement with those of the existing literature, because the FT capability by sector considerably differ. However, we propose that we extend this work in the future by widening our sample (with respect to the number of observations and companies analyzed) so that we can further break down our proposed sectors by activity and outline the results by sub-periods in greater detail, differentiating between boom times and economic crises. In addition, other lines of research that consider different proxy variables, such as the size of the sector analyzed in each case, are also open. Finally, for the companies analyzed in the present study, it would be interesting to investigate whether there is an inverse relationship between FT capability and the sensitivity of returns against changes in interest rates.

To conclude, investor behavior should consider the FT capability of the sector that each company belongs to before making an investment decision. As suggested by previous literature, our results support the state-dependent nature of the investor behavior in the inflation analysis. Similarly, this study may find a herding behavior of investors, because in some scenarios, investors disregard their own information and exhibit herding behavior, which is often extremely optimistic or pessimistic and may lead to an unreasonable reaction to movements in inflation rates when the FTC is higher or lower than the expected one. Finally, we confirm the null hypothesis that investor behavior may depend on different factors that affect the decision-making. Therefore, aspects such as the sector that traded stock belongs to and the business cycle definitely impact on investment behavior. Consequently, the FTC would be a key factor in investment decisions.

## Author contributions

All authors listed, have made substantial, direct and intellectual contribution to the work, and approved it for publication.

### Conflict of interest statement

The authors declare that the research was conducted in the absence of any commercial or financial relationships that could be construed as a potential conflict of interest.

## Footnotes

^1^(*a*) *p* < 0, 15;(*b*) *p* < 0, 10;(*c*) *p* < 0, 05.
