# Supplementary material for: Investor Behavior and Flow-through Capability in the US Stock Market
Source: Front Psychol. 2016 May 9;7:668. doi: 10.3389/fpsyg.2016.00668 (PMC4860425; doi:10.3389/fpsyg.2016.00668)
Supplement: Supplementary file 1 [file Table1.DOCX]

Supplementary Material

Investor Behavior and Flow-Through Capability in the US Stock Market

Carlos Cano1, Francisco Jareño1*, Marta Tolentino2

*** Correspondence:** Corresponding Author: [francisco.jareno@uclm.es](mailto:francisco.jareno@uclm.es)

# Supplementary Figures and Tables

## Supplementary Tables

**Supplementary Table 1. NAICS classification of companies listed in the S&P 500 (2000-2009)**

| **CODE** | **NAICS SECTOR** | **COMPANY S&P 500 ACRONYM** |
| --- | --- | --- |
| 11 | Agriculture, Forestry, Fishing and Hunting | PCL.N |
| 21 | Mining | APC.N, APA.N, BJS.N, COG.N, CHK.N, CNX.N, DNR.N, DVN.N, DO.N, DYN.N, ESV.N, EOG.N, FCX.N, MEE.N, NBR.N, NEM.N, OXY.N, BTU.N, PXD.N, RDC.N, VMC.N, XTO.N |
| 22 | Utilities | AYE.N, AEP.N, AEE.N, CNP.N, CMS.N, ED.N, CEG.N, D.N, DTE.N, DUK.N, EIX.N, ETR.N, EXC.N, FSLR.OQ, FE.N, FPL.N, TEG.N, GAS.N, NI.N, NBL.N, SIN.N, POM.N, PCG.N, PPL.N, PGN.N, PEG.N, STR.N, SCG.N, SIN.N, SO.N, SWN.N, TE.N, AES.N, WEC.N, XEL.N |
| 23 | Construction | DHI.N, FLR.N, HAL.N, JEC.N, KBH.N, LEN.N, PHM.N, PWR.N, SLB.N |
| 31-33 | Manufacturing | MMM.N, ABT.N, AMD.N, A.N, APD.N, AKS.N, AA.N, ATI.N, AGN.N, ALTR.OQ, MO.N, AMGN.OQ, APH.N, ADI.N, AMAT.OQ, AAPL.OQ, ADM.N, AVY.N, AVP.N, BHI.N, BLL.N, BAX.N, BDX.N, BMS.N, BIIB.OQ, BDK.N, BA.N, BMY.N, BRCM.OQ, BFb.N, BCR.N, CAM.N, CPB.N, CAT.N, CELG.OQ, CEPH.OQ, CF.N, CVX.N, CIEN.OQ, CTAS.OQ, CSCO.OQ, CLX.N, CME.OQ, COH.N, CCE.N, KO.N, CL.N, CAG.N, COP.N, STZ.N, GLW.N, CMI.N, DHR.N, DF.N, DE.N, DELL.OQ, XRAY.OQ, DOV.N, DOW.N, DD.N, EMN.N, EK.N, ECL.N, LLY.N, EMC.N, EMR.N, EL.N, XOM.N, FLIR.OQ, FLS.N, FMC.N, FTI.N, F.N, FRX.N, FO.N, GD.N, GE.N, GIS.N, GENZ.OQ, GILD.OQ, GR.N, GT.N, HOG.N, HAR.N, HRS.N, HAS.N, HSY.N, HES.N, HPQ.N, NHZ.N, HON.N, HRL.N, HSP.N, ITW.N, INTC.OQ, IFF.OQ, IGT.N, IP.N, ISRG.OQ, ITT.N, JBL.N, JDSU.OQ, SJM.N, JCI.N, JNJ.N, JNPR.OQ, K.N, KMB.N, KG.N, KLAC.OQ, LLL.N, LEG.N, LXK.N, LLTC.OQ, LMT.N, LSI.N, MRO.N, MAS.N, MAT.OQ, MKC.N, MWV.N, MDT.N, WFR.N, PCS.N, MCHP.OQ, MU.N, MIL.N, MOLX.OQ, TAP.N, MON.N, MOT.N, MUR.N, MYL.OQ, NSM.N, NTAP.OQ, NWL.N, NIKE.N, NOC.N, NVLS.OQ, NOV.N, NUE.N, NVDA.OQ, OI.N, PCAR.OQ, PTV.N, PLL.N, PH.N, PBG.N, PEP.N, PKI.N, PFE.N, PBI.N, RL.N, PPG.N, PX.N, PCP.N, PG.N, QLGC.OQ, QCOM.OQ, RRC.N, RTN.N, RHT.N, RAI.N, ROK.N, COL.N, RRD.OQ, CRM.N, SNDK.OQ, SLE.N, SGP.N, SEE.N, SHW.N, SIAL.OQ, SII.N, SNA.N, SE.N, STJ.N, SWK.N, SYK.N, JAVA.OQ, SUN.N, TLAB.OQ, TDC.N, TER.N, TSO.N, TXN.N, TXT.N, TMO.N, TIE.N, TSN.N, UTX.N, X.N, VLO.N, VAR.N, VFC.N, WAT.N, WPI.N, WDC.N, WY.N, WHR.N, XRX.N, XLNX.OQ, ZMH.N |
| 42 | Wholesale Trade | ARG.N, CAH.N, EQT.N, GPC.N, GWW.N, LO.N, MCK.N, PDCO.OQ, PM.N, SYY.N, WMB.N |
| 44-45 | Retail Trade | ANF.N, AMZN.OQ, AN.N, AZO.N, BBBY.OQ, BBY.N, BIG.N, COST.OQ, CVS.N, FDO.N, FAST.OQ, GME.N, GPS.N, HD.N, JCP.N, KSS.N, KR.N, LTD.N, LOW.N, M.N, MHS.N, JWN.N, ODP.N, ORLY.OQ, RSH.N, SWY.N, SHLD.OQ, SPLS.OQ, SVU.N, TGT.N, TIF.N, TJX.N, WAG.N, WMT.N, WFMI.OQ |
| 48-49 | Transportation and Wharehousing | BNI.N, CCL.N, CHRW.OQ, CSX.N, EP.N, EXPD.OQ, FDX.N, NSC.N, LUV.N, UNP.N, UPS.N |
| 51 | Information | ADBE.OQ, ACS.N, AKAM.OQ, T.N, ADSK.OQ, ADP.OQ, BMC.OQ, CA.OQ, CBS.N, CTL.N, CTXS.OQ, CMCSA.OQ, CPWR.OQ, DTV.OQ, EBAY.OQ, ERTS.OQ, EXPE.OQ, FIS.N, FISV.OQ, FTR.N, GCI.N, GOOG.OQ, INTU.OQ, MFE.N, MHP.N, MDP.N, MSFT.OQ, NYT.N, NWSA.OQ, ORCL.OQ, Q.N, SIN.N, S.N, SYMC.OQ, TWC.N, TWX.N, VRSN.OQ, VZ.N, VIAb.N, DIS.N, WPO.N, WIN.N, YHOO.OQ |
| 52 | Finance and Insurance | AET.N, AFL.N, ALL.N, AIG.N, AXP.N, AMP.N, AOC.N, AIV.N, AIZ.N, AVB.N, BAC.N, BK.N, BBT.N, BXP.N, COF.N, SCHW.OQ, CB.N, CI.N, CINF.OQ, C.N, CMA.N, DFS.N, ETFC.OQ, ETN.N, EQR.N, ESRX.OQ, FII.N, FITB.OQ, FHN.N, BEN.N, GNW.N, GS.N, HIG.N, HCP.N, HCN.N, HST.N, HCBK.OQ, ICE.N, IVZ.N, JNS.N, JPM.N, KEY.N, KIM.N, LM.N, LNC.N, L.N, MTB.N, MMC.N, MI.N, MA.N, MBI.N, MET.N, MS.N, NDAQ.OQ, NTRS.OQ |
| 53 | Real Estate Rental and Leasing | AMT.N, CBG.N, R.N |
| 54 | Professional, Scientific and Technical Services | CTSH.OQ, CSC.N, HRB.N, IBM.N, RX.N, IPG.N, IRM.N, MWW.N, NOVL.OQ, OMC.N, PAYX.OQ |
| 56 | Administrative and Support and Waste Management and Remediation Services | CVG.N, DNB.N, EFX.N, MCO.N, RSG.N, RHI.N, SRCL.OQ, WM.N |
| 61 | Educational Services | APOL.OQ, DV.N |
| 62 | Health Care and Social Assistance | ABC.N, BSX.N, CFN.N, CVH.N, DVA.N, HUM.N, LH.N, LIFE.OQ, DGX.N, THC.N, |
| 71 | Arts, Entertainment and Recreation | PNW.N, WYNN.OQ |
| 72 | Accommodation and Food Services | DRI.N, DPS.N, KFT.N, LUK.N, MAR.N, MCD.N, SBUX.OQ, HOT.N, WYN.N, YUM.N |

Source: Own elaboration based on <http://www.naics.com/search.htm> and <http://www.bloomberg.com/>
