# Supplementary material for: Investor Behavior and Flow-through Capability in the US Stock Market
Source: Front Psychol. 2016 May 9;7:668. doi: 10.3389/fpsyg.2016.00668 (PMC4860425; doi:10.3389/fpsyg.2016.00668)
Supplement: Supplementary file 2 [file Table2.DOCX]

Supplementary Material

Investor Behavior and Flow-Through Capability in the US Stock Market

Carlos Cano1, Francisco Jareño1*, Marta Tolentino2

*** Correspondence:** Corresponding Author: [francisco.jareno@uclm.es](mailto:francisco.jareno@uclm.es)

# Supplementary Figures and Tables

## Supplementary Tables

**Supplementary Table 2. Principal statistics of the variables used in estimating FT coefficients**

**Panel 1: Turnover**

|  | **S1** | **S2** | **S3** | **S4** | **S5** | **S6** | **S7** | **S8** | **S9** | **S10** | **S11** | **S12** |
| --- | --- | --- | --- | --- | --- | --- | --- | --- | --- | --- | --- | --- |
| **Mean** | 0.249832 | 0.304506 | 0.082858 | 0.076483 | 0.039707 | 0.425778 | 0.210698 | 0.093648 | 0.057642 | 0.044676 | 0.038366 | 0.063464 |
| **Median** | 0.193185 | 0.268053 | 0.079828 | 0.091262 | 0.050679 | 0.387160 | 0.301876 | 0.091684 | 0.099892 | 0.090181 | 0.089723 | 0.046790 |
| **Maximum** | 1.421404 | 0.996608 | 0.329485 | 0.183747 | 0.312380 | 3.008.848 | 0.819145 | 0.215891 | 0.196333 | 0.426040 | 0.438327 | 0.813938 |
| **Minimum** | -0.504714 | -0.40434 | -0.26761 | -0.12666 | -0.29466 | -0.687307 | -0.50210 | -0.18841 | -0.30148 | -0.214526 | -0.32664 | -0.49862 |
| **Standard Deviation** | 0.402175 | 0.252975 | 0.112328 | 0.063742 | 0.145112 | 0.611858 | 0.338479 | 0.085177 | 0.118288 | 0.142574 | 0.162094 | 0.256836 |
| **Asymmetry** | 0.612824 | 0.611755 | -0.38900 | -1.60394 | -0.32013 | 1.666250 | -0.35417 | -0.90510 | -1.57835 | 0.080249 | -0.53870 | 0.687791 |
| **Kurtosis** | 3.624150 | 7.531011 | 4.543916 | 5.968779 | 2.273839 | 9.128139 | 2.211631 | 4.389406 | 5.072267 | 3.004166 | 3.346606 | 5.189035 |
| **Observation** | 40 | 40 | 40 | 40 | 40 | 40 | 40 | 40 | 40 | 40 | 40 | 40 |

**Panel 2: Operating costs**

|  | **S1** | **S2** | **S3** | **S4** | **S5** | **S6** | **S7** | **S8** | **S9** | **S10** | **S11** | **S12** |
| --- | --- | --- | --- | --- | --- | --- | --- | --- | --- | --- | --- | --- |
| **Mean** | 0.042059 | 0.064022 | 0.0242940 | 0.018220 | 0.018554 | 0.052263 | 0.043129 | 0.030356 | -0.015451 | 0.0474000 | 0.023115 | 0.021427 |
| **Median** | 0.047300 | 0.064900 | 0.0340000 | 0.022000 | 0.033600 | 0.062100 | 0.043000 | 0.036100 | -0.001700 | 0.0545000 | 0.032200 | 0.020300 |
| **Maximum** | 0.095700 | 0.094200 | 0.0857000 | 0.069900 | 0.124700 | 0.174100 | 0.087900 | 0.109300 | 0.0493000 | 0.1212000 | 0.077100 | 0.083000 |
| **Minimum** | -0.037000 | 0.030200 | -0.061800 | -0.05780 | -0.19030 | -0.203100 | -0.02150 | -0.057900 | -0.160700 | -0.040300 | -0.07000 | -0.02830 |
| **Standard Deviation** | 0.031645 | 0.016852 | 0.041454 | 0.029252 | 0.074371 | 0.093995 | 0.026002 | 0.0458370 | 0.0515890 | 0.0473280 | 0.037221 | 0.027720 |
| **Asymmetry** | -0.431395 | -0.32273 | -0.328282 | -0.97199 | -1.34358 | -0.872891 | -0.42622 | -0.274773 | -1.143600 | -0.2415180 | -0.87838 | 0.277254 |
| **Kurtosis** | 2.746915 | 2.573486 | 1.989150 | 4.000823 | 4.556373 | 3.270790 | 3.035641 | 2.532282 | 3.725630 | 1.8527120 | 3.003602 | 2.196627 |
| **Observation** | 40 | 40 | 40 | 40 | 40 | 40 | 40 | 40 | 40 | 40 | 40 | 40 |

**Panel 3: Number of employees**

|  | **S1** | **S2** | **S3** | **S4** | **S5** | **S6** | **S7** | **S8** | **S9** | **S10** | **S11** | **S12** |
| --- | --- | --- | --- | --- | --- | --- | --- | --- | --- | --- | --- | --- |
| **Mean** | 0.02559 | 0.05495 | -0.00466 | -0.00212 | -0.00485 | -0.02273 | 0.00307 | -0.01667 | -0.03602 | 0.00985 | -0.00101 | -0.00824 |
| **Median** | 0.03740 | 0.05040 | -0.00100 | 0.00330 | 0.00930 | -0.01710 | 0.01650 | -0.01900 | -0.02130 | 0.03060 | 0.00880 | -0.00830 |
| **Maximum** | 0.07890 | 0.09190 | 0.02540 | 0.02660 | 0.07120 | 0.12860 | 0.05160 | 0.07430 | -0.00050 | 0.08765 | 0.02920 | 0.01520 |
| **Minimum** | -0.06930 | 0.02240 | -0.06630 | -0.05420 | -0.17610 | -0.26180 | -0.11350 | -0.06790 | -0.12970 | -0.16270 | -0.06910 | -0.03760 |
| **Standard Deviation** | 0.03986 | 0.01747 | 0.02579 | 0.02086 | 0.06299 | 0.07931 | 0.04117 | 0.04033 | 0.03633 | 0.07199 | 0.02901 | 0.01369 |
| **Asymmetry** | -1.054057 | 0.700455 | -0.82084 | -1.09213 | -1.42138 | -1.16864 | -1.61882 | 0.708773 | -1.09923 | -0.901434 | -0.95210 | -0.17783 |
| **Kurtosis** | 3.020659 | 2.975030 | 2.807397 | 3.56813 | 4.38631 | 5.045484 | 4.850970 | 2.938906 | 3.27566 | 2.771684 | 2.72058 | 2.43548 |
| **Observation** | 40 | 40 | 40 | 40 | 40 | 40 | 40 | 40 | 40 | 40 | 40 | 40 |

S1, S2… S12 identifies the corresponding variable (analyzed in each case) for sectors 1, 2… 12, respectively

**Panel 4: American inflation rate (USIR)**

|  | **USIR** |
| --- | --- |
| **Mean** | 0.024748 |
| **Median** | 0.026980 |
| **Maximum** | 0.063954 |
| **Minimum** | -0.024395 |
| **Standard Deviation** | 0.017391 |
| **Asymmetry** | -0.697027 |
| **Kurtosis** | 4.141.261 |
| **Number of Observations** | 40 |
